# Autophagy Induction as a Host-Directed Therapeutic Strategy against *Mycobacterium tuberculosis* Infection

**DOI:** 10.3390/medicina57060522

**Published:** 2021-05-23

**Authors:** Harresh Adikesavalu, Radha Gopalaswamy, Ashok Kumar, Uma Devi Ranganathan, Sivakumar Shanmugam

**Affiliations:** 1 Department of Bacteriology, ICMR-National Institute for Research in Tuberculosis, Chetpet, Chennai 600031, India; harreshadikesavalu@gmail.com (H.A.); radhagopalaswamy@gmail.com (R.G.); sashokkumarbiotech@gmail.com (A.K.); 2 Department of Immunology, ICMR-National Institute for Research in Tuberculosis, Chetpet, Chennai 600031, India; krumadevi@gmail.com

**Keywords:** *Mycobacterium tuberculosis*, host-directed therapies, autophagy, adjuvants

## Abstract

Tuberculosis (TB), a bacterialinfectious disease caused by *Mycobacterium tuberculosis* (*M.tb*), which causes significant mortality in humans worldwide. Current treatment regimen involve the administration of multiple antibiotics over the course of several months that contributes to patient non-compliance leading to relapse and the development of drug-resistant *M.tb* (MDR and XDR) strains. Together, these facts highlight the need for the development of shorter TB treatment regimens. Host-directed therapy (HDT) is a new and emerging concept that aims to augment host immune response using drugs/compounds with or without adjunct antibiotics against *M.tb* infection. Autophagy is a natural catabolic mechanism of the cell that involves delivering the cytosolic constituents to the lysosomes for degradation and recycling the components; thereby maintaining the cellular and energy homoeostasis of a cell. However, over the past decade, an improved understanding of the role of autophagy in immunity has led to autophagy activation by using drugs or agents. This autophagy manipulation may represent a promising host-directed therapeutic strategy for human TB. However, current clinical knowledge on implementing autophagy activation by drugs or agents, as a stand-alone HDT or as an adjunct with antibiotics to treat human TB is insufficient. In recent years, many reports on high-throughput drug screening and measurement of autophagic flux by fluorescence, high-content microscopy, flow cytometry, microplate reader and immunoblotting have been published for the discovery of drugs that modulate autophagy. In this review, we discuss the commonly used chemical screening approaches in mammalian cells for the discovery of autophagy activating drugs against *M.tb*infection. We also summarize the various autophagy-activating agents, both pre-clinical candidates and compounds approved for advanced clinical investigation during mycobacterial infection. Finally, we discuss the opportunities and challenges in using autophagy activation as HDT strategy to improve TB outcome and shorten treatment regimen.

## 1. Introduction

Tuberculosis (TB) disease continues to be a global health threat with high morbidity and mortality, particularly in developing countries [1]. TB is primarily caused by *Mycobacterium tuberculosis* (*M.tb*), a successful intracellular pathogen that invades human lungs as droplet nuclei [2]. Despite the directly observed treatment short-course (DOTS) program, the incidence of TB is exacerbated by co-infections, co-morbidities, emergence of drug-resistant (DR) *M.tb* strains and a rise in the reservoir of latent *M.tb* infection (LTBI) [3]. The current anti-TB therapy has many limitations including long duration, use of multiple antibiotics, adverse effects of drugs and an associated lack of patient compliance. These limitations highlight the need to develop new treatment and management strategies for both drug-sensitive (DS) and drug-resistant (DR) TB in order to control infection more effectively [4]. The host immune status plays a significant role in TB disease outcome, though *M.tb* possesses several evasion strategies that favors its persistence and survival [5]. Thus, using adjunctive treatments with host-directed therapeutic (HDT) drugs that can modulate the host immune response is a promising strategy to increase the success of TB treatment [4]. Many studies have suggested that autophagy plays a key role in modulating host innate immune response by promoting several critical elements that target and eliminate intracellular pathogens [6,7,8]. Given these observations, the use of HDT drugs to upregulate autophagic pathway is currently receiving considerable attention as it could lead to effective treatment alternatives for both DS and DR TB. In this regard, repurposed compounds with prior safety and regulatory approval that could potentially target autophagy are mostly investigated for further approval as HDT drugs in TB treatment [3].

In this review, we present a brief overview of autophagy in the context of mycobacterial infection, the different methods to monitor and measure autophagic activity and summarize the various HDT drugs targeting autophagy that can be used as an adjunct along with anti-TB drugs (ATDs). Finally, we discuss the challenges associated in using HDT drugs activating autophagy to improve TB treatment outcome. 

## 2. Autophagy—A Brief Overview during Mycobacterial Infection

### 2.1. Autophagy

**Figure 1:**
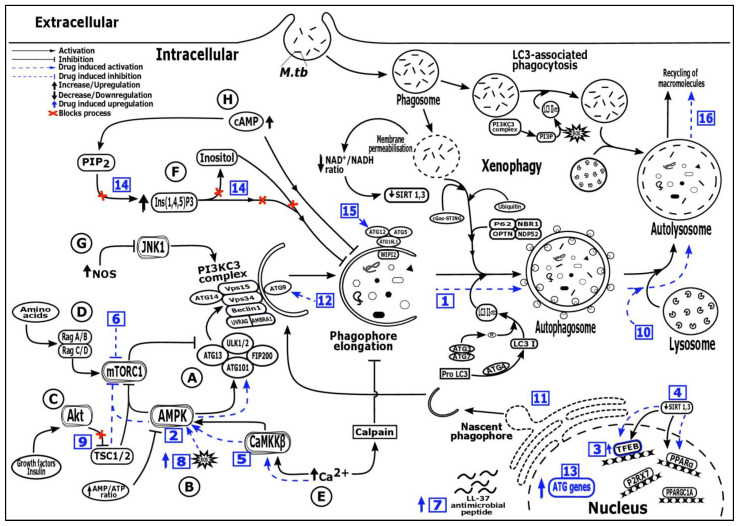
Regulatory mechanism of autophagy pathway and potential targets ofdifferent host directed therapeutic drugs. (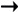): Activation, (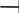):inhibition, (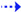): Drug induced activation, (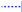): Drug induced inhibition, (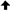): Upregulation, (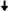): Downregulation, (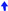): Drug induced upregulation, (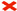): Blocks activation process, Numbers in blue boxes indicate drug induced activation of the following processes—1: Increased autophagosome formation (SMER 18 & 28, nortriptyline); 2: AMPK activation: (AICAR, ornithine, metformin, statins); 3: TFEB signaling upregulation (GSK4112, GW7647, trehalose, Wy14643, honokiol, ambroxol); 4: SIRT 1, 3 activator (SRT 1720, resveratrol, honokiol); 5: Ca^2+^—AMPK signaling (GABA, vitamin D, soyabean lectin, isoniazid, pyrazinamide, calcimycin); 6: mTOR inhibition (Rapamycin, evorilimus, nitazoxanide); 7: LL-37 induced expression (Vitamin D, Interferon-γ, 4-phenylbutyrate); 8: Increased ROS production (Imiquimod); 9: PI3K/Akt/mTOR pathway inhibition (Baicalin, imatinib, nilotinib, ibrutinib, bazedoxifene); 10: Autophagic flux induction (Pasakbumin A, fluoxetine); 11: ER stress mediated autophagy induction (Thiostrepton); 12: Depletion of p38 MAPK mediated autophagy activation via p38IP and mATG9 (Gefitnib); 13: Autophagy genes (ATG16L1 and LC3) upregulation (Loperamide); 14: AMPK activation via inositol signaling pathway (Carbamazepine); 15: mTOR independent autophagy formation via ATG 12 (Valproic acid); 16: Slow autophagic flux and increased lysozyme acidification (Prochlorperazine edisylate), Alphabets (A-H) describes the different pathways—A: mTORC1/ULK/ATG13/FIP200 dependent pathway, B: AMPK/TSC/mTORC1 dependent pathway, C: PI3KC1a/Akt/TSC/mTORC1 dependent pathway, D: Rag/mTORC1 dependent pathway, E: mTOR independent Ca^2+^/calpain pathway, F: mTOR independent inositol signalling pathway, G: mTOR independent JNK1/Beclin1/PI3KC3 pathway, H: mTOR independent cAMP/Ins(1,4,5)P3 pathway, AMBRA1: Activating molecule in BECN1 regulated autophagy protein 1, AMPK: AMP-activated protein kinase, ATG: Autophagy-related genes or proteins, Ca2+: Calcium ions, CaMKKβ: Ca2+/calmodulin-dependent protein kinase kinase β, cAMP/Ins(1,4,5)P3: Cyclic adenosine monophosphate/inositol (1,4,5)-trisphosphate, cGAS-STING: Cyclic GMP-AMP Synthase—simulator of interferon genes, FIP200: focal adhesion kinase family-interacting protein of 200 kDa, JNK1: c-Jun N-terminal kinase 1, LC3: Microtubule associated protein 1 (MAP1) light chain 3, LL-37: Human cathelicidin, NAD+/NADH: Nicotinamide adenine dinucleotide (NAD) + hydrogen (H), NBR1: Neighbor of BRCA1 gene 1, NDP52: Nuclear domain 10 protein 52, NOS: Nitric oxide synthase, OPTN: Optineurin, PE: phosphatidylethanolamine, PI3KC3 complex: Class III phosphatidylinositol 3-kinase (PI3K) complex, PI3KC1a/Akt/TSC/mTORC1: Class 1a phosphoinositide 3-kinase/protein kinase B/tuberous sclerosis complex/mTORC1, PI3P: Phosphatidylinositol 3-phosphate, PIP_2_: Phosphatidylinositol bisphosphate, PPARα: Peroxisome proliferation factor-activated receptor α, PPARGC1A: Peroxisome proliferator-activated receptor gamma coactivator 1-alpha, P2RX7: Purinergic Receptor 7, ROS: Reactive oxygen species, Rag/mTORC1: Ras-related GTP-binding protein/mammalian target of rapamycin complex 1, SIRT: Sirtuin protein, TFEB: Transcription factors EB, ULK1/2: UNC-51-Like Ser/Thr kinase, VPS: Vacuolar protein sorting, WIPI2: WD repeat domain phosphoinositide-interacting protein 2.

Autophagy, discovered in 1963 is a natural catabolic mechanism of the cell in which damaged or dysfunctional cellular components and abnormal protein aggregates are sequestered and fused to lysosomes for degradation. The resultant precursor molecules viz., free amino acids, free fatty acids, and ATPs are recycled back into the cytoplasm for new de novo protein synthesis and energy production [9]. This mechanism is highly conserved in all eukaryotes and plays a pivotal role in maintaining cellular homeostasis under normal conditions [9,10]. There are three main autophagic pathways based on delivery route to the lysosome: microautophagy, macroautophagy and chaperone mediated autophagy among which micro and macro autophagy are found in all eukaryotic cells [11] whereas, chaperone mediated autophagy (CMA) is only identified in mammalian cells [12]. Although these pathways are different from one another, degradation of cytosolic components usually occurs through the lysosomes. In microautophagy, lysosome directly engulfs and degrades small portions of cytosolic substrates for degradation [13]. Alternatively, in CMA cytosolic proteins are translocated into the lysosomal lumen for degradation after being recognized by heat shock cognate protein (HSC70; 71-kDa) containing a KFERQ-like pentapeptide sequence [14,15]. Finally, macroautophagy (henceforth referred to as autophagy) which is totally different from other autophagic pathways where a crescent-shaped isolation membrane or phagophore is formed (Figure 1). This phagophore engulfs the cytosolic components and fuses with itself to form a double-membrane structure termed as autophagosome. Upon maturation, the autophagosome fuses with the lysosome thereby delivering the cargo for degradation by resident hydrolases [16,17,18]. Earlier studies considered autophagy as a non-selective (bulk or generalized) canonical pathway triggered as a survival mechanism in response to stress signals such as nutrient deprivation and hypoxia. However, during the last two decades different studies have shown that autophagy can selectively degrade large endogenous material such as protein aggregates (aggrephagy), lipids (lipophagy), damaged organelles viz., peroxisomes (pexophagy), mitochondria (mitophagy), endoplasmic reticulum (reticulophagy) and ribosomes (ribophagy) as well as exogenous material, such as invading pathogens (xenophagy) through non-canonical pathway which require only a subset of autophagy-related genes [10,19,20,21]. It is evident that apart from cellular homeostasis, autophagy also plays a significant role in modulating host innate immunity by targeting and eliminating intracellular pathogens via xenophagy [6,7].

In the autophagic pathway, the formation of autophagosome, a structurally unique double-membrane organelle is a key characteristic feature. Unlike other cell organelles, autophagosomes can dynamically increase in numbers in cytosol upon receiving stress signals [9]. Classically, autophagy is a canonical pathway that is dependent on a suite of hierarchically organized functional proteins encoded by autophagy-related genes (ATGs) that are regulated by several transcriptional regulators (Figure 1). The core mammalian autophagy related proteins are regulated by transcription factors viz., EB (TFEB), zinc-finger protein with KRAB and SCAN domains 3 (ZKSCAN3), FOXO family of transcription factors (FOXOs), tumor-suppressor protein TP53 (p53), E2 Factor 1 (E2F1) and Nuclear factor kappa B (NF-κB) and peroxisome proliferation factor-activated receptor α (PPARα) [22]. The mechanism of autophagosome formation involves three stages viz., initiation, nucleation, and elongation each of which is mediated by conserved ATGs that are assembled into discrete functional units or complexes at the nascent phagophore. The ULK complex, comprising UNC-51-Like Ser/Thr kinase ULK1/2, ATG13, focal adhesion kinase family-interacting protein of 200 kDa (FIP200) and ATG101, which is controlled by AMP-activated protein kinase (AMPK) and mechanistic target of rapamycin (mTOR). This ULK complex is responsible for the initiation of nascent phagophores at distinct sites in Golgi complex, endoplasmic reticulum (ER) and mitochondria. The class III phosphatidylinositol 3-kinase (PI3K) complex comprises Beclin-1, and AMBRA1 (activating molecule in BECN1 regulated autophagy protein 1) which are released from their inhibitor Bcl-2 via., phosphorylation by the ULK complex, vacuolar protein sorting (VPS15 and VPS34) and ATG14. This complex is involved in the synthesis of phosphatidylinositol-3-phosphate and promotes nucleation by the recruitment of WD repeat domain phosphoinositide-interacting proteins (WIPI) to the nascent phagophore membrane. The transmembrane proteins ATG9, is involved in the supply of lipid bilayers for autophagosome formation. The phagophore is elongated by the action of two ubiquitin-like conjugation complexes viz., ATG12-ATG5-ATG16L1 complex which promotes the second ATG4-ATG7-ATG3 complex and enhances the recruitment of microtubule associated protein 1 (MAP1) light chain 3 (LC3) to the site. The ATG4 cleaves the pro-LC3 to form LC3-I, which is conjugated to phosphatidylethanolamine (PE) associated with the phagophore membrane by ATG7 and ATG3. Finally, the phagophore membrane has elongated and closed onto itself to form autophagosome, resulting in the sequestration of cytosolic content for degradation [8,9,23]. After autophagosome formation, the SNARE protein syntaxin 17 (STX17) localizes and interacts with lysosomal vesicle-associated membrane protein 8 (VAMP8) to form autolysosome, a terminal degradative organelle that enables cargo acidification and hydrolysis [24,25]

Autophagy is governed by numerous signals that are usually categorized into mTOR dependent and independent (Figure 1) [26]. This mTOR protein contains two distinct complexes namely, mTORC1 and mTORC2. The mTOR dependent pathway is a classical pathway that negatively regulates autophagy. The mTORC1, if inhibited by starvation or rapamycin treatment activates ULK1–ATG13–FIP200 complex that signals the autophagic machinery present downstream of mTORC1, resulting in autophagy activation [27,28]. The other signals that activate mTORC1 and suppress autophagy are amino acids which control autophagy through the Ras-related GTP-binding protein/mTORC1(Rag/mTORC1) pathway [29], growth factors which control autophagy through the class 1aphosphoinositide 3-kinase/protein kinase B/tuberous sclerosis complex/mTORC1 (PI3KC1a/Akt/TSC/mTORC1) pathway [30], energy status (high ATP/AMP ratio) and stressors which control autophagy through the AMPK/TSC/mTORC1 pathway [31]. Alternatively, the decrease in amino acids, growth factors and ATP/AMP ratio triggers autophagy activation through the respective pathways. The mTOR independent pathway that negatively regulates autophagy has also been described. The inositol levels, required for inositol signaling pathway inhibits autophagosome synthesis and the inhibition of inositol monophosphatase (IMPase) by pharmacological agents, reduces the levels of free inositol, which results in autophagy activation without inhibiting mTORC1 activity [32]. A change in intracellular Ca^2+^ level and cyclic adenosine monophosphate (cAMP) level regulates autophagy through the Ca^2+^/calpain pathway and cAMP/inositol (1,4,5)-trisphosphate (cAMP/Ins(1,4,5)P3) pathway respectively, in an mTOR independent manner [26,33]. Finally, autophagy is enhanced by the interaction of VPS34, a nutrient dependent lipid kinase (member of the PI3K family) with Beclin-1 through the c-Jun N-terminal kinases/beclin-1/PI3KC3(JNK1/Beclin-1/PI3KC3) pathway in an mTOR independent manner [34].

### 2.2. Autophagy during Mycobacterial Infection

The role of autophagy in pathogen elimination has become increasingly clear over the last two decades [6,8,23]. The intracellular pathogen *M. tuberculosis*, after invading macrophages, resides and replicates within phagosomes and arrests the phagosomal maturation. Eventually, the *M.tb* permeabilize the phagosomal membrane via ESX-1 (Early Secretory Antigenic Target ESAT-6 system 1), a type VII secretion system [35] and escapes into the cytosol. The *M.tb* present in damaged phagosomes and/or in the cytosol triggers autophagy activation through the binding of ubiquitin proteins such as Galectin [36], Parkin [37] and Smurf1 [38]. Additionally, the cytosolic *M.tb* DNA is recognized by the cyclic GMP-AMPSynthase (cGAS)—Stimulator of Interferon Genes (STING) pathway and is tagged by ubiquitin proteins [39]. The tagged *M.tb* and other components are recognized by sequestosome 1/p62-like receptors (SLRs), a subset of autophagic receptors involved in antimicrobial defense. The adaptors P62, neighbor of BRCA1 gene 1 (NBR1), optineurin (OPTN) and nuclear domain 10 protein 52 (NDP52) or calcium binding and coiled-coil domain 2 (CALCOCO2) recognize ubiquitinated *M.tb* and target them to autophagosomes for subsequent degradation [8,23]. Alternatively, *M.tb* phagosomes undergo LC3-associated phagocytosis (LAP), a non-canonical pathway that activates on receiving toll-like receptors (TLR) signaling(Figure 1) [40]. This pathway utilizes certain autophagic proteins which triggers ROS generation and the direct recruitment of LC3 and Beclin-1 to the single-membrane phagosome. The conjugation of lipidated LC3-II to the phagosomal membrane promotes phagosomal maturation and intracellular bacterial killing [41].

Certain innate immune signals such TLRs, cytokine IL-1β and NOD-like receptor 2 (NOD2) are involved in cross talk with autophagic pathways during *M.tb* infection. The TLRs, especially TLR2, TLR4, and TLR9 are responsible for *M.tb* recognition and stimulation by these TLRs induced phagosomal maturation and autophagy activation via myeloid differentiation primary response gene 88 (MyD88), TIR-domain-containing adapter-inducing interferon- β (TRIF), mitogen-activated protein kinase (MAPK) and tumor necrosis factor receptor–associated factor 6 (TRAF6) [42,43,44,45,46]. The cytokine IL-1β induced autophagic killing of *M.tb* in macrophages in a tank binding kinase 1 (TBK-1) dependent manner [47]. Finally, NOD2 that recognizes bacterial molecules (i.e., peptidoglycan) and induces autophagy by upregulating proteins such as LC3, and ATG16L1, resulting in *M.tb* control [48].

### 2.3. Mycobacteria Defense Mechanisms Against Host Immune Response

After entering macrophages, *M.tb* activates mTOR, increases lipid droplets and reduces autophagic capacity of the host in order to protect itself from autophagic elimination [49,50]. Further, a number of *M.tb* bacterial factors such as Eis (enhanced intracellular survival) [51], lipoarabinomannan, a mycobacterial glycolipid [52], ESX-1, a type VII secretion system [35] and *M.tb* protein CpsA [53] have been reported to inhibit host autophagic activity. However, pharmacological agonists of autophagy can overcome anti-autophagy mechanisms of *M.tb* and eventually eliminate *M.tb*.

## 3. Measuring Autophagic Activity

The importance of autophagic activity has increased the need for a sensitive and accurate assay to monitor autophagy with possible high throughput applications. Many earlier reports have characterized several autophagic factors and substrates viz., the ULK1 complex (ULK1, ATG13, FIP200, and ATG101, and ATG9A), double-FYVE domain-containing protein 1 (DFCP1), WIPI family proteins (typically WIPI1), ATG5, ATG16L1, LC3, STX17 and P62/SQSTM1 as markers to study the different stages of autophagy [24,54,55,56,57]. The different methodologies employed to monitor and/or measure autophagic activity by using markers include electron microscopy, fluorescence and high-content image analysis, flow cytometry, immunoblotting and immunoassay [58,59]. In a previous study, Yoshi and Mizushima (2017) have reinforced the importance of evaluating the autophagic flux which includes autophagosome formation, fusion with the lysosomes and cargo degradation in the autolysosomes to accurately monitor and/or measure autophagic activity [60]. The assays for monitoring and measuring autophagy are improving over time to be more sensitive. On this basis, the following chapter describes the commonly employed autophagic markers in various assays to monitor autophagy in mammalian cells.

### 3.1. Monitoring Autophagic Structures

Electron microscopy was used to monitor autophagosomes in the late 1950s, after the discovery of autophagy. However, it was not a perfect method for functional studies as it is difficult to differentiate between the different selective autophagy in mammalian cells. Additionally, the difference between autolysosomes and other endocytic compartments and vacuoles are difficult to differentiate [59].

### 3.2. LC3—A Lipidated Autophagic Protein Marker

The mammalian autophagy protein, LC3 is a widely used marker to study autophagy, since most of the autophagy markers except LC3 homologs, detach either before or after autophagosome formation [9]. The nascent LC3, soon after synthesis is cleaved by ATG4 to form LC3-I that have a glycine residue at the C-terminal end. This cytoplasmic LC3-I, in the presence of ATG7 is post-translationally modified by conjugating with phosphatidylethanolamine at its C-terminal glycine end to form LC3-PE (LC3-II) [61]. Unlike LC3-I that localizes in cytoplasm, the LC3-II associates with autophagosomes both on the outer and inner luminal membrane until maturation that is the fusion of autophagosome and lysosome. After fusion, the lysosomal enzymes degrade the LC3-II on the inner membrane while those on the outer membrane are removed by ATG4 for recycling [9,61]. Many of the assays use LC3 as a substrate reporter to monitor and measure autophagic activity since the amount of LC3-II almost correlates with the autophagosome numbers [59]. Immunoblotting technique can be used to detect the conversion from endogenous LC3-I to LC3-II by using antibodies against LC3. The LC3-II migrates faster on SDS PAGE than LC3-I due to the charged nature of conjugate PE [62,63]. The limitations of this assay are that the amount of LC3-II only indicates autophagosome formation and not the overall autophagic flux. Furthermore, LC3 can also be recruited directly to bacteria-containing phagosome membranes in a process termed LC3-associated phagocytosis and therefore, some amount of LC3-II is from non-autophagosome structures [40,63]. The LC3 turnover assay by western blot or immunoblotting is used to measure autophagic flux by measuring lysosome-dependent LC3-II degradation. The cells are treated with pharmacological inhibitors such as lysosomotropic agents and protease inhibitors that block autophagic flux and prevent LC3-II degradation, resulting in its accumulation. The difference in LC3-II levels in the presence and absence of these agents represents a measure of autophagic flux [64]. It has to be noted that selecting the appropriate lysosomal inhibitors and the ideal concentration is important, as these inhibitors could suppress mTOR activity [65]. Autophagy gene knockdown/knockout can also be used to inhibit autophagy instead of pharmacological agents [66].

The use of a fluorescent-tagged LC3 (GFP-LC3) reporter to study autophagy by immunocytochemistry using fluorescence microscopy is a straightforward approach in which the endogenous GFP-LC3 is observed as GFP-LC3 positive vesicles or punctate structures. The number of punctate structures per cell usually corresponds to autophagosome numbers [67]. The turnover of GFP-LC3 reporters can be quantified in an assay by Fluorescence Activated Cell Sorter (FACS) to measure autophagic activity in living mammalian cells. The disappearance of total LC3, as a result of degradation in autolysosomes, is ideally a good indicator of autophagic flux. Therefore, the amount of total cellular LC3, quantitated by flow cytometry correlates inversely with autophagic flux [68].

Recently, Koepke et al. designed an eGFP-LC3B reporter that can be stably integrated into the target cell and standardized a protocol to remove cytoplasmic eGFP-LC3B-I from cells, resulting in only membrane-bound eGFP-LC3B-II [69]. Although, assays based on LC3 reporters could accurately monitor autophagosome numbers, caution must be exercised due to some potential limitations. This assay fails to differentiate whether the autophagic activity at a given time point is induced or blocked as autophagosome numbers are increased in both the settings. Also, as mentioned above, the possibility of LC3-II localizing on non-autophagosome structures cannot be ruled out. Moreover, LC3 is known to be regulated transcriptionally during autophagy [70] which may lead to wrong interpretation while using LC3 levels as indicators of autophagic flux. Finally, the chance of overestimating the number of autophagosomes is high, since GFP-LC3 punctate dot-like aggregates that do not represent autophagosomes, occur in cells overexpressing GFP-LC3 by transient transfection [67,71,72].

A tandem fluorescent-tagged mRFP-GFP-LC3 reporter can be used to monitor autophagic flux by immunocytochemistry and confocal fluorescence microscopy. This reporter is pH sensitive and the fluorescence of GFP (pKa = 5.9) is quenched in the acidic environment, while mRFP signal is relatively stable at acidic pH. When the LC3 protein localizes to autophagosomes, the reporter exhibits both green and red signals that usually appear as yellow. After the fusion of autophagosome and lysosome, the GFP signal is quenched making autolysosomes appear red. Yellow punctate accumulates, if there is a reduction in autophagic flux (autophagosome–lysosome fusion) and in case of autophagy suppression, both yellow and red punctuate structures are reduced [73]. The limitations of this reporter are that it depends on the acidic activity of the lysosomal enzymes that quenches the GFP signal. Furthermore, the intensity of RFP signal may reduce due to fluorescence reabsorption or fluorescence resonance energy transfer in tandemly fused RFP-GFP probes which may impede accurate quantification. Similarly, another tandem fluorescent-tagged mTagRFP-mWasabi-LC3 can also be used to monitor autophagic flux [74].

Another new probe, GFP-LC3-RFP-LC3ΔG, a fusion protein of GFP-LC3 and RFP-LC3 without C-terminal glycine has been developed to evaluate autophagic flux qualitatively (fluorescence microscopy) as well as quantitatively (microplate reader or flow cytometry). Upon overexpression, this protein is translated as one fusion protein, which is cleaved by ATG4 family proteases, resulting in the same number of GFP-LC3 and RFP-LC3ΔG proteins. During autophagic flux, the GFP-LC3 is degraded while RFP-LC3ΔG remains in cytosol, serving as internal control. Therefore, the autophagic activity can be measured by calculating GFP/RFP ratio that is if the ratio is low then autophagic flux is induced and if the ratio is high, the autophagic flux is blocked. The limitations with this reporter are that homologous recombination could occur between the two LC3 sequences in stable transformants, yielding in GFP-LC3ΔG that cannot be degraded by autophagy. Secondly, the time duration for a clear reduction of the GFP/RFP ratio is high [75].

### 3.3. p62/SQSTM1—An Autophagic Substrate Marker

An alternative approach to evaluate autophagic activity is by monitoring the autophagic degradation of p62/SQSTM1, a known autophagy substrate that functions as an adaptor protein in recruiting specific cytoplasmic components to the autophagosomes [76]. Like LC3, the degradation of endogenous p62 can also be detected by immunoblotting technique in which the decrease in p62 levels indicates autophagic flux activation, whereas the level increases if autophagic activity is blocked or inhibited [77].

The fluorescent tagged GFP-p62 reporter probe can be used to study perturbations in autophagic flux by methods such as immunoblotting, fluorescence microscopy and flow cytometry [78]. In another assay, Min et al. developed a quantifiable luciferase reporter system Luc2p-p62 or Luc2p-p62ΔU to measure autophagic flux. During autophagic flux, the Luc2p-p62 is degraded while Luc2p-p62ΔU remains in cytosol, as a normalization control due to its resistance to autophagic degradation. Therefore, increased Luc2p-p62ΔU/Luc2p-p62 ratio would directly reflect autophagic flux induction and vice versa [79].

A time-resolved fluorescence resonance energy transfer (TR-FRET) assay has been described which uses both LC3B-II and p62 markers to monitor autophagic activity. This method relies on energy transfer between two fluorophores, a donor that transfers energy when excited and an acceptor that emits a fluorescent signal, when situated in close proximity. The increased autophagic activity results in increased LC3-II signal and decreased p62 signal, whereas when autophagic flux is blocked at the lysosome fusion stage both LC3-II and p62 signals are increased [80]. It must be noted that, like LC3 transcriptional upregulation has also been reported and therefore autophagic flux may need to be measured by a combination of other techniques [79].

### 3.4. Other Markers to Monitor Autophagic Activity

In addition, to LC3 and p62, early markers of autophagy viz., ATG5 [81], ATG16 [82], DFCP1 [83], and WIPI-1 [84] can be assessed to monitor the different stages of autophagic activity. Further, the phosphorylation of ATG14-Ser29 can also be used to monitor autophagy initiation [85]. Tian et al. developed and characterized a monoclonal antibody to monitor newly forming autophagosomes by measuring phosphorylation of the endogenous protein ATG16L1. The described antibody can be used in western blot, immunofluorescence, and immunohistochemistry. Since it is present during autophagy initiation, its levels are not affected by late-stage autophagy blocks and can discriminate very well between autophagy induction and autophagosome accumulation [86].

Though several different markers and assays to monitor autophagic activity have been reported, there is still no single “gold standard” assay to measure autophagic activity in mammalian cells [66]. The techniques available to measure autophagy ranges from direct observation and measurement of autophagosomes in a cell using electron microscopy to more specific quantification of protein markers, primarily LC3 and p62/SQSTM1. Improvised techniques, including the use of fluorescent reporters like GFP, RFP or luciferase reporter like Luc2 to measure autophagic markers have superior accuracy than conventional immunoblotting methods. These systems aid in high throughput assays using either fluorescent microscopy, flow cytometry, microtiter plate assays or TR-FRET assays. However, measuring autophagic flux, which corresponds to the amount of autophagic degradation in a cell, is crucial in determining autophagy accurately. In addition to measuring autophagic flux, it is highly recommended to use secondary assays for additional characterization. The upregulation of different autophagy proteins at the mRNA level may not necessarily correlate with functional autophagy, nonetheless, it can reflect an intact signaling pathway. Therefore, measuring mRNA levels of autophagy proteins by PCR-based approaches can still be used as a secondary assay to characterize perturbations in autophagic activity [87]. When a decrease in autophagic flux is reported, the efficiency of autophagosome–lysosome fusion can be determined by estimating colocalization between autophagosomal (LC3-II, STX17) and lysosomal (Lysosomal-associated membrane protein 1) markers [88]. Galectins can be used to evaluate endo-lysosomal damage caused by pathogens and lysomotrophic agents [89]. Further, lysosomal activity can be evaluated by using LysoTracker which monitors lysosomal acidification [88] and lysosomal-METRIQ which monitors the overall lysosomal integrity [90].

## 4. Potential Autophagy Activating Drugs for Host Directed Therapy against Mycobacterial Infection in Pre-Clinical Trials

Host directed therapeutic strategy that enhances the protective immunity against emerging infectious diseases has gained significant importance over the last two decades [91]. Host directed therapeutic drugs as adjuncts with existing TB drugs for *M.tb* infection could lead to shorter and more effective treatments for tuberculosis. A literary search of pre-clinical trials and animal model studies revealed that repurposing licensed drugs with autophagy inducing potential, showed effective therapeutic manipulation of host immunity against *M.tb* infection. Many of these drugs already have well-defined safety and pharmacokinetic profiles and are more likely to be investigated in randomized and controlled clinical trials that will evaluate their effectiveness in TB. The potential HDT drug candidates from different drug/compound types that target autophagy and the mechanism involved in manipulating host immunity against *M.tb* infection are summarized in Figure 1 and Table 1.

### 4.1. Small-Molecules

Small molecules are increasingly being tested for their ability to enhance autophagy against different disease phenotypes [92]. Many small-molecules have also been reported for their ability to inhibit intracellular *M.tb* replication through autophagy activation [93,94,95,96,97,98,99,100,101]. Flotoet al. described two compounds termed as small-molecule enhancers of rapamycin (SMERs) that induce autophagy at the stage of autophagosome formation without decreasing mTOR activity. The SMER 18 and 28 had the highest autophagic activity in *M.tb* infected human peripheral blood mononuclear cells (PBMC), resulting in the inhibition and clearance of intracellular *M.tb* [93].

The small molecule 5-aminoimidazole-4-carboxamide-1-β-D-ribofuranoside (AICAR), a direct AMPK activator, was reported to enhance autophagy in different mammalian cells viz., human monocytic cell line (THP-1 cells), RAW 264.7 cells and mice bone marrow derived macrophages (BMDMs) against *M. bovis* BCG and *M. tuberculosis* strain H37Rv [94]. This molecule induces autophagy in *M.tb* infected cells by activating AMPK that inhibits *M.tb*-mediated mTOR activation. Furthermore, this AMPK activation by AICAR induced Peroxisome proliferator-activated receptor gamma coactivator 1-alpha (PPARGC1A), a transcriptional coactivator protein that upregulated multiple autophagy related genes via CCAAT/enhancer-binding protein β (CEBPB) and enhanced autophagic flux in *M.tb* infected cells [94].

The synthetic small molecule, GSK4112 that acts as an agonist to nuclear receptor subfamily 1, group D, member 1 (NR1D1), a transcriptional repressor protein was reported to induce autophagic flux and increase lysosome biogenesis [95]. The transcriptional protein NR1D1 plays a key role in infection and inflammation [102] and its activation led to the modulation in the expression of transcription factor EB (TFEB). The upregulation of TFEB increased the number of both autophagosomes and lysosomes in *M. tuberculosis* strain H37Rv infected THP-1 cells [95].

Kim et al. reported that GW7647, a synthetic small molecule agonist of the PPARα transcription factor enhances autophagic flux against *M. bovis* BCG and *M. tuberculosis* strain H37Rv in mice BMDMs. The PPARα activation by GW7647 resulted in the upregulation and translocation of TFEB, a critical regulator of various genes involved in autophagic flux. Further, PPAR-α activation inhibited lipid body formation during mycobacterial infection [96].

The small molecule SRT 1720, a synthetic activator of sirtuin 1 (SIRT1) deacetylase was observed to restrict the growth of intracellular mycobacteria in THP-1 cells and human primary monocyte-derived macrophages(HMDMs) [97]. The SIRT1 mainly regulates cellular homeostasis, and its activity depends on the availability of intracellular nicotinamide adenine dinucleotide (NAD+) [135]. Cheng et al. reported that the *M.tb* infection reduced the intracellular NAD+/NADH ratio resulting in the down-regulation of SIRT1 expression. This was reversed by the addition of SRT 1720 and in addition, the SRT 1720 mediated SIRT1 activation in mycobacteria infected cells induced autophagy and phagosome-lysosome fusion [97].

The small molecule NSC 18725, a pyrazole derivative was reported to inhibit intracellular *M.tb* growth by inducing autophagy in differentiated THP-1 macrophages [98].

The amino acid Gamma-aminobutyric acid (GABA), a potent neurotransmitter inhibitor, was linked to autophagy activation and host protection against intracellular mycobacterial infections [99]. Autophagy was promoted in macrophages on treatment with GABA. The GABAergic treatment in *M.tb* infected macrophages triggered the increase in intracellular Ca^2+^ levels and phosphorylation of AMPK, resulting in autophagic activation. Further, GABAergic activation increased the expression of GABARAPL1, a key autophagy-associated protein required for phagosomal maturation [99].

Mouse alveolar macrophages (AMs) supplemented with amino acid ornithine was reported to enhance autophagy resulting in increased *M.tb* clearance [100]. Generally, ornithine plays a crucial role in disposing ammonia produced in cells through deamination of amino acids via urea cycle [100]. Mycobacteria also produce and utilize ammonia as a source of nitrogen for its metabolic activity in infected macrophages [136]. Therefore ornithine supplementation in *M.tb* infected AMs reduced ammonia levels and additionally upregulated AMPK phosphorylation which inhibits mTOR resulting in autophagy activation [100].

Trehalose, a naturally occurring disaccharide was reported to facilitate autophagy in different cell lines (U937, U1.1 and HEK293T) against *M.tb* and non-tuberculous mycobacterial (NTMs) strains either alone or during co-infection with HIV-1 [101]. This disaccharide small molecule induced autophagic flux by increasing phosphatidylinositol 3,5-bisphosphate (PtdIns(3,5)P2) that served asmucolipin subfamily, member 1(MCOLN1) channel agonist and increased Ca^2+^ release from lysosomal lumen [137]. The released Ca^2+^ activates calcineurin, a serine-threonine phosphatase that dephosphorylates TFEB, resulting in trehalose mediated nuclear translocation of TFEB and mTOR independent autophagy activation in macrophages [101]. Additionally, trehalose caused a pseudo-starvation like response by competitively inhibiting GLUT transporters viz., SLC2A3/GLUT3 and SLC2A8/GLUT8, resulting in autophagy induction via mTOR inhibition and AMPK activation [101].

### 4.2. Immunosuppressants

Rapamycin a macrolide immunosuppressive compound and its analog everolimus were reported to enhance autophagy through mTOR inhibition and concomitantly suppress the growth of intracellular *M.tb* and *M. bovis* BCG strains in different cells [6,103].

### 4.3. Immunomodulators

Vitamin D plays an important role as an immunomodulator in strengthening the innate immune system to fight against pathogens [138]. In addition, vitamin D was reported to enhance autophagic flux in macrophages and restrict the growth of intracellular *M.tb* [104,105]. The active form of vitamin D that is 1,25-dihydroxyvitamin D3 (1,25D3) increased autophagic flux by inducing the gene expression of human antimicrobial protein, cathelicidin (hCAP-18/LL-37) which in turn triggers MAPKs and C/EBP β-binding sites, resulting in transcriptional activation of Beclin-1 and ATG5. Vitamin D also promoted cathelicidin recruitment into *M.tb* containing autophagosome through calcium/calmodulin dependent protein kinase-β (CAMKK-β) and AMPK dependent pathways in infected macrophages [104,105]. The cytokine interferon-γ (IFN-γ) was studied for its ability to induce autophagic flux and inhibit intracellular *M.tb* in different cells viz., human T cells, primary human monocytes and HMDMs.

The IFN- γ mediated activation of autophagic flux in *M.tb* infected cells was dependent on vitamin D sufficiency [106].

Imiquimod, a nucleoside analog of imidazoquinoline that stimulates TLR7 was reported to enhance autophagy and control *M.tb* infection in Raw264.7 cells and THP-1 cells. The imiquimod mediated autophagy activation was associated with induced oxidative stress, triggered by mitochondrial reactive oxygen species (ROS) production that activates selective autophagy (mitophagy) by enhancing interaction between Beclin-1 and BCL2/adenovirus E1B 19 kDa protein-interacting protein 3 (BNIP3), resulting in intracellular *M.tb* elimination in infected macrophages. Further, imiquimod also upregulated the nitric oxide (NO) production by the mitogen activated protein kinase/extracellular-signal-regulated kinase 1/2 (MEK/ERK1/2) and glycogen synthase kinase 3 β (GSK-3β) signaling pathways that lead to autophagy induction [107].

Lipopolysaccharides (LPS), an endotoxin derived from the outer membrane of gram-negative bacteria that functions as a TLR 4 agonist was reported to induce autophagy as evidenced by the significant rise in protein expression in *M.tb* infected THP-1 cells [108].

### 4.4. Plant Compounds

Many plant compounds have been studied for their ability to induce autophagy against *M.tb* infection [97,109,110]. Resveratrol, a stilbene derivative, was reported to activate SIRT1 deacetylase that lead to the induction of autophagy and phagosome-lysosome fusion in *M.tb* infected THP-1 and HMDMs cells [97].

Baicalin, a flavone glycoside, induced autophagy by inhibiting the PI3K/Akt/mTOR signaling pathway in *M.tb* infected macrophages. Additionally, baicalin-mediated autophagy activation inhibited *M.tb* infection caused nuclear factor kappa B (NF-kB) signaling, NLR family pyrin domain containing 3 (NLRP3) inflammasome activity and the production of pro-inflammatory cytokine IL-1b [109].

Pasakbumin A, a eurycomanone compound isolated from *Eurycoma longifolia,* restricted the growth of *M.tb* strain H37Rv in different cells such as Raw264.7, and THP-1 cells [110]. This compound activated both autophagy and tumour necrosis factor alpha (TNF-α) production through the ERK1/2-mediated signaling pathway. Further, pasakbumin A induced phagosomal maturation in *M.tb* infected macrophages [110].

Epigallocatechin-3-gallate (EGCG), a major polyphenolic compound found in green tea extract, was reported to enhance autophagic flux and suppress intracellular growth of *M.tb* in Raw264.7 cells and mice [111].

Honokiol, a low molecular weight polyphenols that activates sirtuin 3 (SIRT3) deacetylase, was reported to promote autophagic functions in different macrophages viz., BMDMs, HMDMs and human peripheral blood mononuclear cells (PBMCs) against *M. bovis* BCG and *M.tb* H37Rv strains [112]. The honokiol-mediated autophagy activated SIRT3, which consecutively triggered the expression of PPARα transcription factor resulting in the upregulation and translocation of TFEB, a critical regulator of various genes involved in autophagic flux [112].

Soybean lectin (SBL) isolated from soybean (*Glycine max*) seeds was reported to induce autophagy and curtail intracellular growth of *M. smegmatis* mc^2^ 155 and *M. bovis* BCG in THP-1 cells [113]. The SBL activated P2RX7 receptor that triggered the downstream activation of Ca^2+^—AMPK signaling pathway. Further, the activated P2RX7 triggered ROS generation via NF-κB activation and nuclear translocation, resulting in autophagic flux activation [113].

### 4.5. Antibiotics

Antimycobacterial drugs, viz., isoniazid (INH) and pyrazinamide (PZA), were studied for their ability to activate autophagy in human and murine macrophages against *M.tb* infection. The antibiotic induced autophagy was triggered by the increased mitochondrial ROS production via the enzyme NADPH oxidase 2 (NOX2). In addition, these antibiotics induce intracellular calcium influx that triggers Ca^2+^ dependent autophagy through the downstream phosphorylation of AMPK [114].

Thiostrepton, a thiopeptide antibiotic, was reported to induce autophagy in *M. marinum*infected RAW264.7 cells and a zebrafish model. This antibiotic inhibits proteasomes that leads to an increase in unfolded/misfolded proteins resulting in ER stress, followed by selective autophagy activation as a defense mechanism for cell survival [115].

Calcimycin, a polyether antibiotic from *Streptomyces chartreusensis*, was reported to kill intracellular mycobacteria by inducing autophagy in infected THP-1 cells. This drug binds to P2RX7 receptor and induces autophagy by triggering the upregulation of intracellular Ca^2+^ levels, resulting in AMPK phosphorylation and increasing interleukin—12 (IL-12) production via NF-κB activation [116,117].

### 4.6. Steroids

Dehydroepiandrosterone (DHEA) is a steroid hormone that activates PPARα and enhances autophagic flux in THP-1 cells infected with *M. tuberculosis* [118].

### 4.7. Anti-Cancer Drugs

Anti-cancer drugs are used to either destroy or slow the growth of cancer cells. These drugs are given with a curative intent or as a palliative therapy that aims to reduce symptoms and prolong life [139]. Apart from cancer treatment, some anticancer drugs were also evaluated for their ability to induce autophagy [119,120,121,122,123,124]. Stanley et al. reported that gefitinib, a signal transduction inhibitor that inhibits epidermal growth factor receptor (EGFR) induces autophagy in a number of cell lines including THP-1 cells, RAW 264.7 cells, and J774A.1 cells against *M.tb* strain H37Rv. This drug restricts *M. tuberculosis* growth in cells by depleting p38 mitogen activated protein kinase (p38 MAPK) molecules in EGFR/p38 MAPK signaling pathway. As p38 MAPK acts as a negative regulator of autophagy, its depletion activates autophagy viap38-interacting protein(p38IP) and mATG9 [119].

In another study, 4-phenylbutyrate (PBA), a histone deacetylase inhibitor that activates transcription activation via acetylation of histones, was reported to induce autophagy in HMDMs and THP-1 cells against *M.tb* strain H37Rv [120]. This drug induced the expression of the antimicrobial peptide LL-37 that activates an mTOR-independent autophagic pathway via the purinergic receptor P2RX7 which signals the downstream activation of AMPK and PI3K in the presence of intracellular Ca^2+^ [120].

Imatinib, a tyrosine kinase inhibitor that increases autophagic flux by activating cathepsin D and phagolysosomal acidification, results in the inhibition of intracellular *M.tb* infection in human macrophages [121]. Nilotinib, a tyrosine kinase inhibitor, was identified to regulate autophagy and inhibit *M. bovis* and *M. avium* subspecies *paratuberculosis* (MAP) in different cells such as THP-1 cells, RAW264.7 cells and BMDMs. Nilotinib inhibits the phosphorylation of PI3k/Akt/mTOR signalling by blockingabelson tyrosine kinase (c-ABL) in mycobacteria infected macrophages [122]. Hu et al. [123] reported that the drug ibrutinib, which inhibits Bruton’s tyrosine kinase (BTK) suppresses intracellular *M.tb* growth by inducing autophagy in THP-1 cells via inhibition of BTK/Akt/mTOR pathway. By inhibiting BTK, ibrutinib blocks the downstream signaling molecule protein kinase Cβ (PKC β) which is an essential regulator of Akt/mTOR signaling pathway that suppresses autophagy [140]. Additionally, ibrutinib also facilitates the completion of autophagic flux that degrades intracellular *M.tb* in autolysosome compartments [123]. Bazedoxifene, a newer selective estrogen receptor modulator was reported to inhibit *M.tb* growth significantly in THP-1 cells by inducing autophagy. This drug increases mROS production and promotes autophagosome formation via phosphorylation of Akt/mTOR signaling [124].

### 4.8. Anti-Diabetic Drugs

Anti-diabetic drugs are used in the treatment of type 2 diabetes mellitus, a metabolic condition mainly characterized by hyperglycemia [141]. Metformin is an antidiabetic drug of the biguanide class that functions by inhibiting mitochondrial complex I which leads to increased cytoplasmic ADP:ATP and AMP:ATP ratios and the activation of AMPK enzyme [142]. This drug was reported to restrict the growth of intracellular BCG as well as the H37Rv strain of *M.tb* in THP-1 cells and HMDMs by enhancing autophagic flux [125].

### 4.9. Anti-Diarrheal Drugs

Antidiarrheal drugs are used to treat acute or chronic diarrhea by binding to opiate receptors in the gastrointestinal tract, resulting in decreased peristaltic activity [143]. Loperamide, a synthetic opioid–phenylpiperidine derivative commonly used for the treatment of diarrhea, was reported to restrict intracellular growth of *M. tuberculosis* in different macrophages viz., murine alveolar macrophages, human alveolar macrophages and HMDMs. This drug induced autophagy in *M.tb* infected macrophages by increasing the upregulation of ATG16L1 and LC3 gene expression and the induced autophagy pathway was completed as evidenced by autophagic substrate p62 degradation [126].

### 4.10. Anti-Protozoal Drug

Nitazoxanide, a thiazolide class of antiprotozoal drug, stimulates autophagy and inhibits intracellular *M. tuberculosis* proliferation in different cell lines such as THP-1, Michigan cancer foundation-7 (MCF-7), human embryonic kidney cells(HEK) 293T and mouse embryo fibroblasts (MEF) cells [127]. This drug inhibited human quinone oxidoreductase NQO1, a scavenger for a broad range of reactive substrates including the ROS. The authors speculate that NQO1 inhibition by nitazoxanide may increase oxidative stress, resulting in mTORC1 inhibition and autophagy activation, thereby inhibiting intracellular *M.tb* proliferation [127].

### 4.11. Anti-Seizure Drugs

Anti-seizure drugs are generally prescribed to patients with epilepsy, a condition that causes recurrent seizures [144]. Additionally, antiseizure drugs from anticonvulsant class are also used for the treatment of several non-epileptic neurological conditions and psychiatric disorders [145]. Some of the first-generation classic anticonvulsants viz., carbamazepine and valproic acid were studied for their ability to induce autophagy [128]. Carbamazepine, a sodium channel blocker that binds and inactivates voltage-gated sodium channels, which inhibits receptors of the central nervous system (CNS). Schiebleret al. reported that carbamazepine induces antimicrobial autophagy against *M. bovis* BCG and *M.tb* strain H37Rv in both mammalian cells and animal models. This drug induces AMPK activation of autophagy by an mTOR-independent pathway, which is controlled by cellular depletion of myo-inositol levels [128]. Schiebleret al. also worked with valproic acid, a drug that inhibits GABA transaminase and increases GABA levels in CNS. This drug induces mTOR-independent autophagy by increasing the rate of autophagosome formation in *M.tb* infected cells [128].

### 4.12. Lipid Lowering Drugs

Lipid lowering drugs are used in the treatment of hyperlipidemia that functions by decreasing the production and increasing the degradation of cholesterol levels [146]. Nonetheless, some drugs from the group fibrates and statins were evaluated for their ability to induce autophagy [96,129,130,131,132]. The fibrate Wy14643 enhanced autophagic flux in *M.tb* infected mice BMDMs by inducing the transcription factor PPARα, resulting in the upregulation and nuclear translocation of TFEB [96]. Alternatively, statins are also a group of lipid lowering drugs used in the treatment of hypercholesterolemia, which functions by inhibitingβ-hydroxy β-methylglutaryl-CoA (HMG-CoA) reductase [146]. Studies have reported that statins such as simvastatin, rosuvastatin and atorvastatin enhanced autophagy and phagosomal maturation in different cells viz., against *M. leprae*, *M. bovis* and *M.tb* infection [129,130,131,132]. Statins decrease cholesterol levels and alter cellular AMP:ATP ratios in *M.tb* infected macrophages, resulting in the activation of autophagy via AMPK-mTORC1-TFEB axis. Further, the statin mediated decrease in intracellular cholesterol levels had induced phagosomal maturation and lysosomal fusion in *M.tb* infected macrophages [129].

### 4.13. Mucoactive Drugs

Mucoactive drugs are used to treat mucus hypersecretion, a clinical complication in respiratory diseases [147]. Ambroxol, a mucokinetic drug that suppresses excessive mucus secretion, was described to enhance autophagic flux through the activation of TFEB nuclear translocation in *M.tb* infected BMDMs and also in mice model [133].

### 4.14. Psychotropic Drugs

Psychotropic drugs are generally prescribed to patients with mental disorders [148] nonetheless; some psychotropic drugs from antidepressant and antipsychotic classes were evaluated for their ability to induce autophagy [119,134]. Nortriptyline, an antidepressant drug that functions by inhibiting norepinephrine and serotonin reuptake, was reported to induce autophagy in both HeLa cells and HMDMs against *M. bovis* BCG, *M.tb* strain H37Rv and two different clinical isolates. Nortriptyline modulates autophagy by increasing the rate of autophagosome formation [134]. Similarly, another antidepressant drug fluoxetine that inhibits the reuptake of serotonin was reported to enhance autophagy and increase the level of TNF-α in different cells viz., THP-1 cells, RAW264.7 cells and BMDMs against *M.tb* strain H37Rv [119]. Prochlorperazine edisylate, an antipsychotic drug that functions by inhibiting postsynaptic dopamine receptor, was observed to impair intracellular survival of mycobacteriaby modulating autophagy in both HeLa cells and HMDMs. This drug reduces autophagic flux and increases the acidity of lysosomes which results in a concomitant reduction in intracellular mycobacteria [134].

## 5. Host Directed Therapeutic Drugs Tested as Adjuncts for Tuberculosis in Clinical Trials

Although many drugs are reported with HDT potential against *M.tb* infection only few drugs were tested for their potential to act as adjuncts with existing TB drugs [97,98,110,125,132,133,149,150]. The details ofdifferent host directed therapeutic drugsin clinical trialsthat are used for the treatment of tuberculosisare summarized in Table 2. The administration of metformin in combination with either INH or ethionamide (ETH), enhanced the efficacy and decreased *M.tb* load in the lungs of infected mice [125]. An ongoing randomized clinical trial (Trial registration number: CTRI/2018/01/011176) evaluates the safety and efficacy of metformin as an adjunct with rifampicin (RIF) ATD in patients with new, smear-positive pulmonary tuberculosis [151]. According to the National Institutes of Health (NIH), clinicaltrials.gov resource database, a trial has recruited adults to evaluate safety, pharmacokinetics and effects of imatinib on myelopoiesis when given as an adjunct with ATDs viz., rifabutin, PZA, INH and ethambutol (EMB) (NCT03891901). The drug SRT 1720, when administered in combination with INH containing antituberculosis therapy, increased the efficacy of INH as demonstrated by decreased bacillary loads in the lungs of *M.tb* infected mice [97]. The small molecule NSC 18,725 was observed to synergize with INH and improve the MIC_99_ value of INH by 4.0-fold against *M.tb* in a checkerboard assay. On the other hand, this molecule when combined with other ATDs viz., RIF, EMB, Bedaquiline (BDQ), BTZ043, and PA-824 it showed only additive effects [98]. The plant compound pasakbumin A, when tested in combination with ATDs viz., RIF and INH reduced the intracellular *M.tb* growth in infected RAW264.7 cells [110]. The mucokinetic drug ambroxol when administered in combination with RIF, increased the anti-mycobacterial effects of RIF in *M.tb* infected mice. In addition, this combination increased the level of RIF in mice lung tissue. Contrastingly, ambroxol in combination with PZA was mildly antagonistic with no increase in antimycobacterial effects of PZA [133]. The statin drug atorvastatin, when combined with rifampin showed a synergistic effect and reduced intracellular mycobacterial viability both in THP-1 cells and mice model [132]. Similarly, Dutta et al. [149] reported yet another statin drug, simvastatin that significantly enhanced bactericidal activity against *M.tb* infection when combined with first-line ATDs viz., INH, RIF and PZA in both THP-1 cells and mice model [149]. Based on the clinicaltrials.gov resource database, three clinical trials investigating the efficacy, safety, tolerability, pharmacokinetics, and time to culture conversion of different statin drugs viz., atorvastatin (NCT04721795) rosuvastatin (NCT04504851) and pravastatin (NCT03882177) as adjunctive therapy for TB are active. A total of 19current and completed trials of vitamin D either as HDT or as dietary supplementation in TB are listed on the clinicaltrials.gov database (Table 2). However, differences in trial outcomes have impeded the interpretation about the efficacy of vitamin D as HDT for TB. For instance, the clinical trial (NCT00419068) that studied whether vitamin D enhances response to standard antibiotic treatment failed to affect time to sputum culture conversion [152]. Contrarily, another clinical trial (NCT01580007) that studied the efficacy of PBA and vitamin D3 as adjunctive host directed therapy for tuberculosis resulted in better clinical recovery to standard short course TB therapy [150]. Lastly, a clinical trial (NCT02968927) was conducted in South Africato study the efficacy of multiple drugs such as everolimus (an analog of rapamycin), CC-11050, vitamin D3 and auranofin (an organogold compound) as adjunctive host directed TB therapies for TB. The study does confirm that CC11050 and everolimusshows safety and tolerability as well as improved forced expiratory volume (FEV) indicating a potential benefit to current TB treatment [153,154].

Although preliminary findings from these clinical trials have shown promising results for the use of HDT in TB treatment, most HDTdrugs are used as supplements and indicate an indirect role in autophagy induction. In one clinical trial (NCT01580007), where use of PBA and vitaminD_3_ as adjunct to standard ATDs were studied, autophagy induction was studied in ex vivo macrophages. Immunofluorescence showed LC3 expression in a specific group that received PBA and vitamin D_3_in addition to standard ATDs. The same group of patients showed faster sputum conversion [155]. However, there is a huge challenge in the selection of dose and duration of treatment for various HDTdrugs that are used as adjuncts to standard ATDs. In the different clinical trials of vitamin D_3_, various doses were usedgiven for different durations. In addition, HDTdrugs like auranofin and ergocalciferol showed severe adverse effects, warranting caution in the dose and duration of HDTdrug treatmentwhen combined with standard ATDs. Therefore, additional clinical studies with more subjects are needed to determine the dose and duration of treatment before these HDTdrugs can be implemented as adjuncts to standard ATDs for TB therapy.

## 6. Challenges in Studying Autophagy Activating Host Directed Therapeutic Drugs to Improve TB Treatment

The identification of drugs that target autophagic flux without affecting other upstream signaling pathways is a challenging task. As pointed out by Paik et al. [167], the information obtained from all the previous studies on drugs for HDT against TB focused only on autophagy activation for inhibiting *M.tb* survival in host cells and failed to discuss the off-target and associated side effects, if any, by these autophagymodulating drugs. Additionally, the risk of TB reactivation in LTBI individuals and increased disease progression as a result of HDT strategies is another challenge that requires attention [4].

Although several drugs that induce autophagy have been reported, the data on some of these drugs are still insufficient. Since, certain assays such as monitoring lysosome activity by LysoTracker, acridine orange, or monodansylcadaverine (MDC) and ATG mRNA or protein expression levels are not accepted as appropriate indicators for monitoring autophagy. In addition, different assays that study autophagosome accumulation as indicators of autophagic induction may not be accurate, since a block in autophagosomal maturation also increases autophagosome numbers [59]. As described above, different techniques are available to study autophagic activity; however, each technique has its own limitations that are different from one another. On this basis, evaluating autophagic activity using a combination of assays rather than a single one would yield reliable information on both autophagosome accumulation and rate of autophagic flux induction. However, choosing the appropriate combination of assays and when to perform it is still a challenging task [59,66,168]. Mizushima and Murphy discussed another important challenge that is to identify biomarkers for autophagy and develop assays that could be used for applied research and clinical trials to measure autophagic flux in humans [66].

Malherbe et al. conceptualized that during active disease mycobacteria could exist at different states of replication and treating this full TB spectrum with a single HDT drug would definitely be a challenge [169]. Further, the HDT drug treatment for TB patients may vary depending on the prognosis that is either active or latent infection and also on the health status of the individual [3]. Therefore, enrolling appropriate patients with good compliance is an important factor, when evaluating HDT drugs as adjuncts options for treating TB. Finally, unknown factors such as appropriate HDT drug dosage, treatment duration, genetic polymorphisms in target receptor and targeted drug delivery to avoidunwanted side effects are some notable challenges in studying autophagy activating HDT drugs as adjunct treatment options for TB [3,4,167].

## 7. Conclusions

The search for new treatment strategies for better management of TB is still underway. HDT is a potential strategy that primarily modulates the impaired host immune responses, which in turn controls bacterial infection. Autophagy activation by drugs or compounds has gained importance for its role in *M.tb* elimination during infection. However, the mechanisms controlling these responses are not completely understood. Identifying the best assay that can be used in high-throughput screening applications is yet another challenge for which investigations are still in progress. The many methods described above have its own strengths and weaknesses and therefore, the ideal recommendation is to choose a combination of independent assays based on the aim of the study in order to avoid false interpretations. Future studies on new and improved assays to measure autophagy will greatly enable investigators to understand the fundamental mechanisms clearly and it also favors the development of autophagy based HDT.

Although the use of HDT drugs targeting autophagy to improve TB outcome is a promising strategy, even for controlling drug-resistant strains, according to in vitro and preclinical studies current data on its clinical use are still insufficient. Moreover, autophagy targeting HDT drugs as an adjunct strategy for the treatment of *M.tb* is still in its infancy. Therefore, future clinical studies should be carefully designed with selected inclusion and exclusion criteria and implemented in order to validate the efficiency of existing HDT drugs as adjuncts with chemotherapeutic drugs and to identify new HDT agents that target autophagy in TB treatment.

## Figures and Tables

**Table 1:** List of potential host directed therapeutic agents targeting autophagy and their mechanism to aid antimycobacterial host defense.

Class	Drugs/Compounds	Drug Action	Mechanism of Autophagy Activation during Mycobacterial Infection	Model	Reference
**Small Molecules**					
SMER	SMER18 and 28	-	Induced autophagosome formation	Human PBMCs	[93]
Analog of AMP	AICAR	Allosteric activation of AMPK kinase which plays a key function in cellular homeostasis	Activates AMPK-PPARGC1A pathway that upregulates CEBPB-dependent autophagy genes and enhances autophagy.	RAW264.7 cells, THP-1 cells (human monocytic cell line), BMDMs, mice and *Drosophila*	[94]
Synthetic small molecule	GSK4112	Activates NR1D1 receptor	Increases autophagic flux via upregulation of TFEB signaling	THP-1 cells, primary human monocyte, murine macrophage cell line, RAW264.7, HEK293T and HepG2 cell lines.	[95]
	GW7647	Activates PPARα receptor	Increases autophagic flux via upregulation of TFEB signaling, and enhanced lipid catabolism	BMDMs	[96]
	SRT 1720	SIRT 1 activator	Enhances autophagy by activating SIRT 1	THP-1 cells, HMDMs and mice	[97]
	NSC 18725	Anti-mycobacterial activity	Modulates autophagy, mechanism unknown	THP-1 cells	[98]
Amino acid	Gamma amino Butyric acid	Neurotransmitter inhibitor	Increases autophagic flux via Ca^2+^-AMPK signaling pathway. Additionally, increases phagosomal maturation	Human PBMCs, HMDMs, RAW264.7 cells and BMDMs	[99]
	Ornithine	Crucial role in disposing excess nitrogen (ammonia) via urea cycle	Increases autophagy by reducing ammonia levels there by upregulating AMPK phosphorylation	Mouse alveolar macrophage, peritoneal macrophages, kupffer cells and BMDMs	[100]
Disaccharides	Trehalose	-	Induces autophagic flux by increasing PI(3,5)P2 levels that activates calcineurin triggered translocation of TFEB. Additionally, it causes a pseudo-starvation like response by inhibiting glucose transporters (GLUT 3 and 8) to induce autophagy	U937, U1.1 and HEK293T cell lines	[101]
**Immunosuppressants**					
Macrolide compound	Rapamycin	Forms an immunosuppressive complex by binding to the immunophilin and also a potent mTOR inhibitor	Autophagy induction via mTORC1 complex inhibitor	Raw264.7 cells, HMDMs, Human PBMCs and BMDMs	[6]
Rapamycin analog	Everolimus *	Inhibits the activation of mTOR by forming a complex with FKBP-12 protein	Autophagy induction via mTORC1 complex inhibitor	-	[103]
**Immunomodulators**					
Vitamin	Vitamin D *	Regulation of hormone secretion, cell proliferation, differentiation and immune response	Induces autophagic flux via a signaling cascade that is triggered by the induced expression of human cathelicidin (hCAP-18/LL-37)	Primary human monocytes, HMDMs, THP-1 cells and RAW 264.7 cells	[104,105]
Cytokine	Interferon-γ (IFN-γ)	Promotes macrophage activation	Activates autophagic flux through vitamin D dependent effector pathway	Human T cells, primary human monocytes and HMDMs	[106]
Nucleoside analog of imidazoquinoline, a synthetic tricyclic organic molecule	Imiquimod	TLR7 and 8 agonist	Induces Autophagy by increasing mitochondrial ROS that triggers selective autophagy. Additionally, upregulates NO Production via the MEK/ERK1/2 and GSK-3β mediated Pathways.	Raw264.7 cells and THP-1 cells	[107]
Endotoxin derived from the outer membrane of Gram-negative bacteria	Lipopolysaccharides (LPS)	TLR4 agonist	Activates autophagy and restores *M.tb* inhibited immune activity	THP-1 cells	[108]
**Plant compounds**					
Stilbene	Resveratrol	SIRT 1 activator	Enhances autophagy by activating SIRT 1	THP-1 cells, HMDMs and mice	[97]
Flavone glycoside	Baicalin	-	Induces the activation of autophagy by inhibiting PI3K/Akt/mTOR pathway. Additionally, inhibits the PI3K/Akt/NF-kB signal pathway, thereby limiting the NLRP3 inflammasome and subsequent production of pro-inflammatory cytokine IL-1β	Mice, raw264.7 cells, murine macrophage	[109]
Eurycomanone	Pasakbumin A	-	Induces autophagic flux and TNF-α production via activation of the ERK1/2-signaling pathway and enhances phagosome maturation and lysosome fusion	Raw264.7 cells, and THP-1 cells	[110]
Polyphenolic compound	Epigallocatechin gallate	-	Induces autophagic flux	Raw264.7 cells and mice	[111]
Lignans (low molecular weight polyphenols)	Honokiol	SIRT 3 activator	Increases autophagic flux via upregulation of TFEB signaling	Mice, BMDMs, HMDMs and Human PBMCs	[112]
Legume Lectins	Soybean lectin	-	Induces autophagic flux by activating P2RX7 that triggers Ca^2+^/AMPK signaling pathway and ROS generation via P2RX7/NF-κB axis	THP-1 cells	[113]
**Antibiotics**					
Small molecule–Isonicotinic acid derivative	Isoniazid	Inhibits the enzyme inh A during mycolic acid synthesis	Induces autophagic flux via NOX- derived ROS and calcium, Ca^2+^ and AMPK dependent pathways	BMDMs and HMDMs	[114]
Small molecule—Nicotinamide analogue	Pyrazinamide	Disrupts membrane potential, interferes with energy production and inhibits trans-translation by binding to ribosomal protein S1	Induces autophagic flux via NOX- derived ROS and calcium, Ca^2+^—dependent AMPK activation	BMDMs and HMDMs	[114]
Thiopeptide	Thiostrepton	Disrupts prokaryotic translation by inhibiting the dissociation of elongation factor G from ribosomes	ER stress mediated autophagy activation	Zebrafish and Raw264.7 cells	[115]
Polyether	Calcimycin	Forms stable complexes with divalent cations and helps in membrane transportation	Induces autophagic flux by activating P2RX7 that triggers Ca^2+^/AMPK signaling pathway and IL-12 generation via P2RX7/NF-κB axis	THP-1 cells	[116,117]
**Steroids**					
Hormones	Dehydroepiandrosterone	Inhibits voltage-gated T-type calcium channels and activates PPARα	Induction of autophagy	THP-1 cells	[118]
**Anticancer drugs**					
Signal transduction inhibitor	Gefitinib	EGFR inhibitor	Enhancing host autophagy by inhibiting EGFR-mediated phosphorylation of the downstream signaling molecule p38 MAPK. Depletion of p38 MAPK activates autophagy via p38IP and mATG9	J774 macrophages and BMDMs	[119]
Histone deacetylase inhibitor	4-phenylbutyrate *	Transcription activation via acetylation of histones	LL-37-mediated autophagy activation via P2RX7 receptor which in turn activates AMPK and PI3K downstream of the P2RX7 receptor together with enhanced cytosolic free Ca2^+^	HMDMs, and THP-1 cells	[120]
Kinase inhibitor	Imatinib *	Tyrosine kinase inhibitor	Increases autophagic flux by activating cathepsin D and increasing phagolysosomal acidification via the inhibition of ABL tyrosine kinase	Human PBMCs, HMDMs, human alveolar macrophages	[121]
	Nilotinib	Tyrosine kinase inhibitor	Promotes autophagy by inhibiting the ABL tyrosine kinase mediated PI3K/Akt/mTOR pathway	THP-1 cells, RAW264.7 cells and BMDMs	[122]
	Ibrutinib	Bruton’s tyrosine kinase (BTK) inhibitor	Induces autophagy through inhibition of BTK/Akt/mTOR pathway and also facilitates the completion of autophagic flux	THP-1 cells	[123]
Estrogen agonists	Bazedoxifene	Selective estrogen receptor modulator	Enhances autophagosome formation via phosphorylation of Akt/mTOR signaling	THP-1 cells	[124]
**Antidiabetic drugs**					
Biguanides	Metformin *	Activates AMPK via inhibiting mitochondrial respiratory complex I which elevates 5’-adenosine monophosphate (AMP) levels	Increases autophagic flux via enhancing autophagosome—lysozome fusion and additionally increases mROS production	THP-1 cells, HMDMs and mice	[125]
**Antidiarrheal drugs**					
Synthetic opioid—phenylpiperidine derivative	Loperamide	Decreases peristaltic activity by binding to opiate receptors in gastrointestinal tract, blocks voltage-dependent calcium channel and calmodulin inhibitor	Increased autophagy induction by upregulating the expression of genes viz., ATG16L1 and LC3	Mice, HMDMs, murine alveolar cells and Human alveolar macrophages	[126]
**Antiprotozoal agents**					
Antiprotozoals	Nitazoxanide	Inhibits pyruvate:ferredoxin oxidoreductase enzyme-dependent electron transport and disrupts metabolism in anaerobic microbes	Autophagy induction via mTORC1 complex inhibitor	THP-1 cells, MCF-7 cells, HEK 293T cells and MEF cells	[127]
**Antiseizure drugs**					
First-generation (classic) anticonvulsants	Carbamazepine	Inactivates Na+ channels and inhibits receptors of CNS	Induction of mTOR-independent autophagy through Ins(1,4,5)P3depletion and AMPK activation	RAW264.7 cells, HMDMs, human alveolar macrophages, zebrafish and mice	[128]
	Valproic acid	Inhibits GABA transaminase and increases GABA levels in CNS. It also inhibits histone deacetylase	Induction of mTOR-independent autophagosome formation through ATG12	RAW264.7 cells, HMDMs and human alveolar macrophages	[128]
**Lipid-lowering drugs**					
Fibrate	Wy14643	Activates PPARα receptor protein	Increases autophagic flux via upregulation of TFEB signaling, and enhanced lipid catabolism	Mice and BMDMs	[96]
Statins	Pravastatin *, Rosuvastatin *, Atorvastatin * and Simvastatin	HMG-CoA reductase inhibitors	Promotes autophagy via the AMPK/mTORC1/TFEB axis. Additionaly increases phagosome maturation and lysosome fusion	Human PBMCs, HMDMs, THP-1 cells and mice	[129,130,131,132]
**Mucoactive drug**					
Mucokinetics	Ambroxol	Suppresses excessive mucus secretion by inhibiting NO-dependent activation of soluble guanylate cyclase	Induction of autophagy via, the activation of TFEB nuclear translocation	Mice and BMDMs	[133]
**Psychotropic Drugs**					
Anti-depressant	Nortriptyline	Norephinephrine and sereotonin reuptake inhibitor	Induces the formation of autophagosomes	HeLa cells and HMDMs	[134]
	Fluoxetine	Sereotonin reuptake inhibitor	Induces autophagy by increasing the secretion of TNF- α	THP-1 cells, RAW264.7 cells, J774 macrophages and BMDMs	[119]
Antipsychotics	Prochlorperazine edisylate	D2 dopamine receptor inhibitor	Slows down autophagic flux and progressively increases the acidity of lysozymes	HeLa cells and HMDMs	[134]

*: Clinical trial information are detailed in Table 2, ABL: Abelson tyrosine kinase, AICAR: 5-aminoimidazole-4-carboxamide-1-β-D-ribofuranoside, AMPK: Adenosine monophosphate kinase, BMDMs: Bone marrow derived macrophages, Ca^2+^: Calcium ions, CNS: Central nervous system, EGFR: Epidermal growth factor receptor, ER: Endoplasmic reticulum, FKBP-12: FK binding protein-12,GSK-3β: Glycogen synthase kinase 3 β, HEK 293T:Human embryonic kidney 293Tcells, HMDMs: Human primary monocyte-derived macrophages, HMG-CoA: β-Hydroxy β-methylglutaryl-CoA,HepG2: Human hepatoma cell lines, IL-12: Interleukin—12,Ins(1,4,5)P3: inositol (1,4,5)-trisphosphate, LL-37: Human cathelicidin (hCAP-18/LL-37), MCF-7: Michigan cancer foundation-7, MEF: Mouse embryo fibroblasts, MEK/ERK1/2: Mitogen activated protein kinase/extracellular-signal-regulated kinase 1/2, mTOR: Mammalian target of rapamycin, NLRP3: NLR family pyrin domain containing 3, NO: Nitric oxide, NR1D1: Nuclear receptor subfamily 1, group D, member 1, PBMCs: Peripheral blood mononuclear cells, p38 MAPK: p38 mitogen activated protein kinase, p38IP: p38-interacting protein, PI3K/Akt/mTOR: Phosphoinositide 3-kinase/protein kinase B/mammalian target of rapamycin, PI3K/Akt/NF-kB: Phosphoinositide 3-kinase/protein kinase B/Nuclear factor kappa B, PI(3,5)P2: Phosphatidylinositol 3,5-bisphosphate, PPARα: Peroxisome proliferator-activated receptor-alpha, PPARGC1A: Peroxisome proliferator-activated receptor gamma coactivator 1-alpha, P2RX7: Purinergic Receptor 7, ROS: Reactive oxygen species, SIRT: Sirtuin protein, SMER: Small molecule enhancers of rapamycin, TFEB: Transcription factor EB, TLR: Toll-like receptor, TNF-α: Tumor necrosis factor-α.

**Table 2:** Summary of the different host directed therapeutic drugs in clinical trialsfor the treatment of tuberculosis *.

S No	Trial ID	Host Directed Therapeutic Drugs	HDT Drug Doseage (mg)	HDT Treatment Duration	Anti-Tuberculosis Drugs (ATD) (Dose)	Study Title	Phase	No. of Participants	Ages Eligible for Study	Study Sponsor	Country	Status	Remarks/Findings
1	CTRI/2018/01/011176	Metformin	1000 mg	Given daily for the first 2 months together with ATD followed by another 4 months with only ATD	Rifampicin, isoniazid, ethambuthol and pyrazinamide (Standard doseage)	Evaluation of metformin in combination with rifampicin containing antituberculosis therapy in patients with new, smear-positive pulmonary tuberculosis (METRIF)	2	316	18–60 Years	National Institute for Research in Tuberculosis	India	Active	Not applicable
2	NCT 03891901	Imatinib	50 mg, 100 mg, 200 mg and 400 mg	Daily for 14 days followed by another 14 days together with ATD	Isoniazid (300 mg) and rifabutin (300 mg)	A Clinical Trial of the Safety, Pharmacokinetics and Hematologic Effects of Imatinib on Myelopoiesis in Adults When Given With and Without Isoniazid and Rifabutin (IMPACT-TB)	2	72	18 to 55 Years	National Institute of Allergy and Infectious Diseases (NIAID)	United States, georgia	Recruiting	Not applicable
3	NCT 04721795	Atorvastatin	30–40 mg	Daily for 2 months together with ATD followed by another 4 months with only ATD	Rifampicin, isoniazid, ethambuthol and pyrazinamide (Standard doseage)	Treating Tuberculosis With the Lipid Lowering Drug Atorvastatin in Nigeria (ATORvastatin in Pulmonary TUBerculosis) (ATORTUB)	2	150	18 to 65 Years	Obafemi Awolowo University Teaching Hospital			
4	NCT 04504851	Rosuvastatin	10 mg	Daily for 2 months together with ATD followed by another 4 months with only ATD	Rifampicin (10 mg/Kg), isoniazid (5 mg/Kg), ethambuthol (25 mg/Kg) and pyrazinamide (15 mg/Kg)	Rosuvastatin Evaluation as a Tuberculosis Treatment Adjunct (ROSETTA)	2	154	18 to 75 Years	National University Hospital, Singapore	Philippines, Singapore, Uganda and Vietnam	Not yet recruiting	Not applicable
5	NCT 03882177	Pravastatin	40 mg, 80 mg, 120 mg and 160 mg	Given alone on 1st day followed by another 14 days together with ATD	Rifampicin, isoniazid, ethambuthol and pyrazinamide (Standard doseage)	StAT-TB (Statin Adjunctive Therapy for TB): A Phase 2b Dose-finding Study of Pravastatin in Adults With Tuberculosis	2	35	18 Years and older	National Institute of Allergy and Infectious Diseases (NIAID)	South Africa	Active, not recruiting	Not applicable
6	NCT 02968927	Everolimus	0.5 mg	Daily for 112 days together with ATD followed by another 68 days with only ATD	Rifabutin (Standard doseage)	A Ph2 Randomized Trial to Evaluate the Safety Preliminary Efficacy and Biomarker Response of Host Directed Therapies Added to Rifabutin-modified Standard Therapy in Adults With Drug-Sensitive Smear-Positive Pulmonary TB	2	200	18 to 65 Years	The Aurum Institute NPC	South Africa	Active, not recruiting	Everolimus in adults as adjunctive therapy for tuberculosis was safe and also improved recovery [154]
7	NCT 00918086	Vitamin D	1.25 mg	Three times a week for a total of 8 weeks followed by another 8 weeks with the same dose given every other week as dietary supplement	Standard ATDs	Impact of Vitamin D Supplementation on Host Immunity to Mycobacterium Tuberculosis and Response to Treatment	2	199	18 Years and older	Emory University	United States, Georgia	Completed	Vitamin D supplementation failed to improve the rate of sputum Mtb clearance [156]
8	NCT 01722396	Vitamin D	2.5 mg	Given 8 weeks apart at 8, 16 and 24 weeks as dietary supplement together with standard ATD	Standard ATDs	Pharmacogenetics of Vitamin D Supplementation in Tuberculosis	3	62	16 Years and older	University of Birmingham	United Kingdom	Completed	Result awaited
9	NCT 00788320	Vitamin D	1.25 mg	Three times a week for a total of 8 weeks as dietary supplement	Standard ATDs	Antimicrobial Peptide LL-37 (Cathelicidin) Production in Active Tuberculosis Disease: Role of Vitamin D Supplementation	NA	0	18 Years and older	Atlanta VA Medical Center	United States, Georgia	Withdrawn (Inadequate enrollment)	Not applicable
10	NCT 04593524	Vitamin D	0.025 mg	4 weeks	Standard ATDs	The Role of Vitamin D, A, and Beta Carotene in Tuberculosis Patients With Vitamin D Receptor Gene Polymorphism	NA	48	20 to 60 Years	Universitas Sumatera Utara	Indonesia	Completed	Vitamin D supplementation to patients with vitamin D receptor gene polymorphism showed increased sputum conversion rates.
11	NCT 00507000	Cholecalciferol (vitamin D)	1.5 mg	Given weekly for 2 months followed by the same dose per month for the next 4 months as dietary supplement	Standard ATDs	Role of Oral Vitamin D as an Adjunct Therapy in Category I Pulmonary Tuberculosis Along with Assessment of Immunological Parameters.	3	150	18 to 60 Years	Indian Council of Medical Research	India	Unknown	Not applicable
12	NCT 01130311	cholecalciferol (vitamin D)	15 mg	Given at week 0 and week 4 as dietary supplement	Standard ATDs	Clinical Trial of Vitamin D Replacement in Patients With Pulmonary Tuberculosis (SUCCINCT)	NA	259	15 Years and older	Aga Khan University	Pakistan	Completed	Vitamin D supplementation showed improved recovery in all TB patients. It also increased host immune activation in vitamin D deficient patients [157]
13	NCT 01244204	Vitamin D	0.020 mg	Daily dose of 800IU of vitamin D	Standard ATDs	Vitamin D Supplementations as Adjunct to Anti-tuberculosis Drugs	NA	120	10 to 18 Years	Harvard School of Public Health	Mongolia	Completed	Vitamin D supplementation resulted in fewer tuberculin skin test conversions [158]
14	NCT 00677339	Vitamin D	1.25 mg	Given once per month as dietary supplement	Standard ATDs	L-arginine and Vitamin D Adjunctive Therapy in Pulmonary Tuberculosis (TB) (AVDAPT)	3	200	15 Years and older	Menzies School of Health Research	Indonesia	Completed	Vitamin D supplementation showed no effect on TB outcomes [159]
15	NCT 01698476	Vitamin D	0.125 mg	Given twice daily for 16 weeks as dietary supplement	Standard ATDs	Immune Reconstitution in Tuberculosis Disease Using Antimicrobial Treatment With Vitamin D and Phenylbutyrate	2	390	18 to 75 Years	Karolinska Institutet	Ethiopia	Completed	Daily supplementation along with PBA results in reduction of clinical TB symptoms while the intervention had no effect on sputum conversion [160]
16	NCT 01698476	4-phenylbutyrate(PBA)	500 mg	Given twice daily for 16 weeks	NA	Immune Reconstitution in Tuberculosis Disease Using Antimicrobial Treatment With Vitamin D and Phenylbutyrate	2	390	18 to 75 Years	Karolinska Institutet	Ethiopia	Completed	Daily supplementation together with vitamin D results in reduction of clinical TB symptoms while the intervention had no effect on sputum conversion [160]
17	NCT 02169570	Vitamin D	15 mg	Given at week 0, 4 and 12 as dietary supplement together with standard ATD	NA	Effect of Supplementary Vitamin D in Patients With Diabetes Mellitus and Pulmonary Tuberculosis (EVIDENT Study)	4	435	30 to 60 Years	Dow University of Health Sciences	Pakistan	Unknown	Not applicable
18	NCT 01580007	Vitamin D	0.125 mg	Given once daily for 2 months	NA	Clinical Trial of Phenylbutyrate and Vitamin D in Tuberculosis (TB)	2	288	18 to 60 Years	International Centre for Diarrhoeal Disease Research, Bangladesh	Bangladesh	Completed	Vitamin D supplementation together with standard short-course therapy showed improved clinical recovery and better sputum culture conversion [150,155]
19	NCT 01580007	4-phenylbutyrate(PBA)	500 mg	Given twice daily for 2 months	NA	Clinical Trial of Phenylbutyrate and Vitamin D in Tuberculosis (TB)	2	288	18 to 60 Years	International Centre for Diarrhoeal Disease Research, Bangladesh	Bangladesh	Completed	PBA supplementation together with vitamin D results in improved clinical recovery and better sputum culture conversion [150,155]
20	NCT 03011580	Vitamin D3	0.240 mg	Given every day for 8 weeks as dietary supplement	NA	Vitamin D3 to Enhance Resolution of Residual Pulmonary Inflammation in Patients Completing Antituberculosis Treatment (ResolveD-TB)	2	15	20 Years and older	Queen Mary University of London	United Kingdom	Completed	Result awaited
21	NCT 01657656	Vitamin D	3.5 mg	Given twice a week as dietary supplement together with standard ATD	NA	Vitamin D Supplementations as Adjunct to Anti-Tuberculosis Drugs in Mongolia	NA	350	18 to 80 Years	Harvard School of Public Health	Mongolia	Completed	Vitamin D supplementation had no effect on sputum culture conversion [161]
22	NCT 01992263	Vitamin D	0.015 mg, 0.050 mg and 0.100 mg	Given Daily for 12 months as dietary supplement	NA	A Trial of Vitamin D Supplementation Among Tuberculosis Patients in South India	NA	200	18 to 60 Years	Cornell University	United States and India	Not yet recruiting	Not applicable
23	NCT 00366470	Vitamin D	2.5 mg	Given once every two weeks for 2 Months as dietary supplement together with standard ATD	NA	A Clinical Trial to Study the Effect of the Addition of Vitamin D to Conventional Treatment in New Pulmonary Tuberculosis Patients	3	250	18 to 75 years	Peter Daley	India	Completed	Vitamin D supplementation showed no reduction in time to sputum culture conversion [162].
24	NCT 02276755	Cholecalciferol (vitamin D3)	0.35 mg	Given weekly for 3 years as dietary supplement	NA	Vitamin D in TB Prevention in School Age Children	3	8851	6 to 13 Years	Harvard School of Public Health	Mongolia	Completed	Vitamin D supplementation failed to lower risk of tuberculosis infection [163]
25	NCT 02880982	Cholecalciferol (vitamin D3)	0.25 mg	Given weekly for 3 years as dietary supplement	NA	Trial of Vitamin D Supplementation in Cape Town Primary Schoolchildren (ViDiKids)	3	1743	6 to 11 Years	Queen Mary University of London	South Africa	Active, not recruiting	Not applicable
26	NCT 00419068	Cholecalciferol (vitamin D3)	2.5 mg	Given at week 0, 2, 4 and 6 as dietary supplement together with standard ATD	NA	Trial of Adjunctive Vitamin D in Tuberculosis Treatment	3	146	18 Years and older	Barts &The London NHS Trust	United Kingdom	Completed	Vitamin D supplementation showed no effect on time to sputum culture conversion in the whole study population. However, participants with the known vitamin D receptor polymorphism showed quicker sputum culture conversion [152]
27	NCT 00157066	Ergocalciferol (vitamin D)	2.5 mg	Single dose as supplement	NA	Effects of Vitamin D Supplementation on Antimycobacterial Immunity	NA	230	18 Years and older	Barts & The London NHS Trust	United Kingdom	Completed	Improved in vitro restriction of BCG-lux luminescence was observed. In addition, antigen-stimulated IFN-gamma was not affected [164]

* As per theICMR—National Institute of Medical Statistics website and US-NIH clinical trial website accessed on 13 May 2021 [165,166].

## Data Availability

Not applicable.
